# Supplementary material for: Prognostic Potential of Cancer-Associated Fibroblast Surface Markers and Their Specific DNA Methylation in Prostate Cancer
Source: Diagnostics (Basel). 2025 Sep 24;15(19):2434. doi: 10.3390/diagnostics15192434 (PMC12524081; doi:10.3390/diagnostics15192434)
Supplement: Supplementary file 1 [file diagnostics-15-02434-s001.zip › Figure S1.pdf]

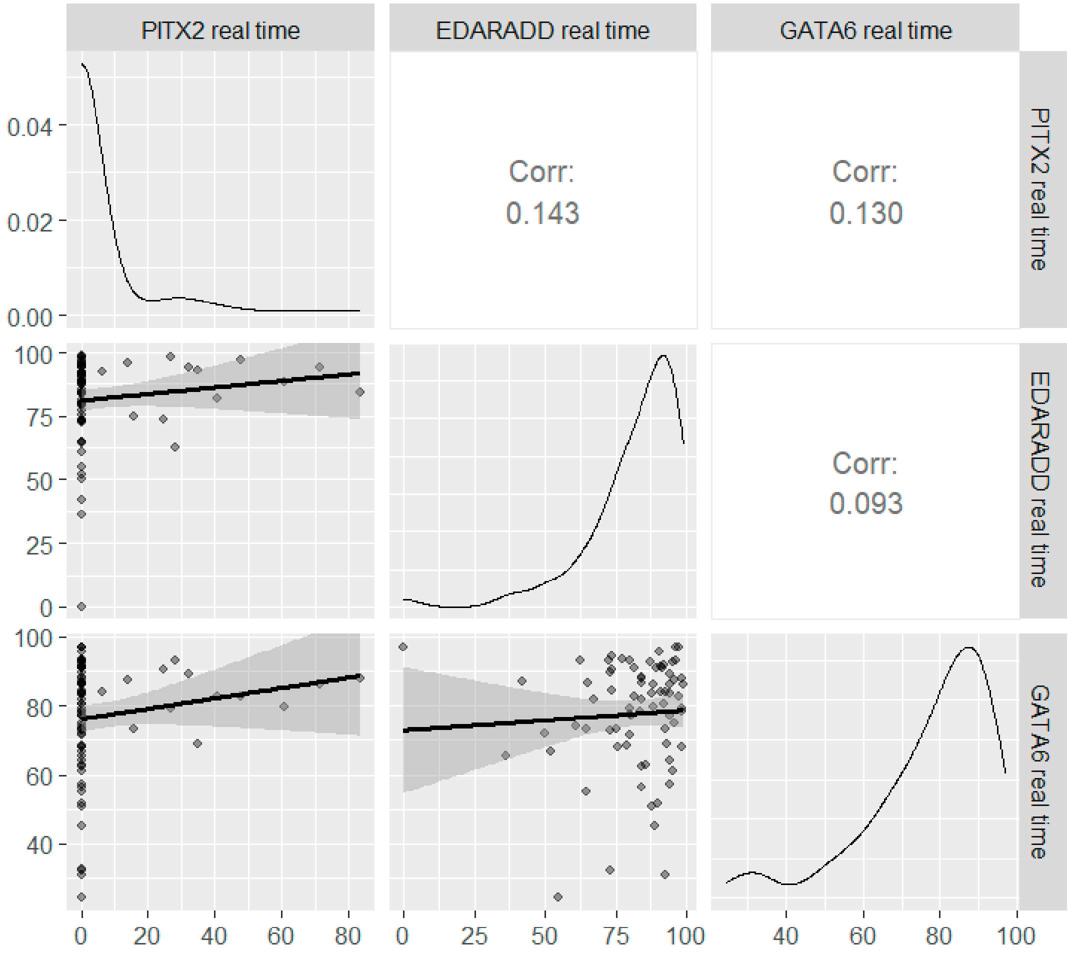

Figure S1. Correlation analysis for DNA methylation profiles in different genes using real-time PCR. The upper right panels show the correlation between the DNA methylation profiles in different genes, the lower left panels show the scatter plots of the DNA methylation profiles in different genes, the diagonal panels show the density plots of the DNA methylation profiles in different genes. All data presented as methylation levels (%).
