# Supplementary material for: Prognostic Potential of Cancer-Associated Fibroblast Surface Markers and Their Specific DNA Methylation in Prostate Cancer
Source: Diagnostics (Basel). 2025 Sep 24;15(19):2434. doi: 10.3390/diagnostics15192434 (PMC12524081; doi:10.3390/diagnostics15192434)
Supplement: Supplementary file 1 [file diagnostics-15-02434-s001.zip › Figure S2.pdf]

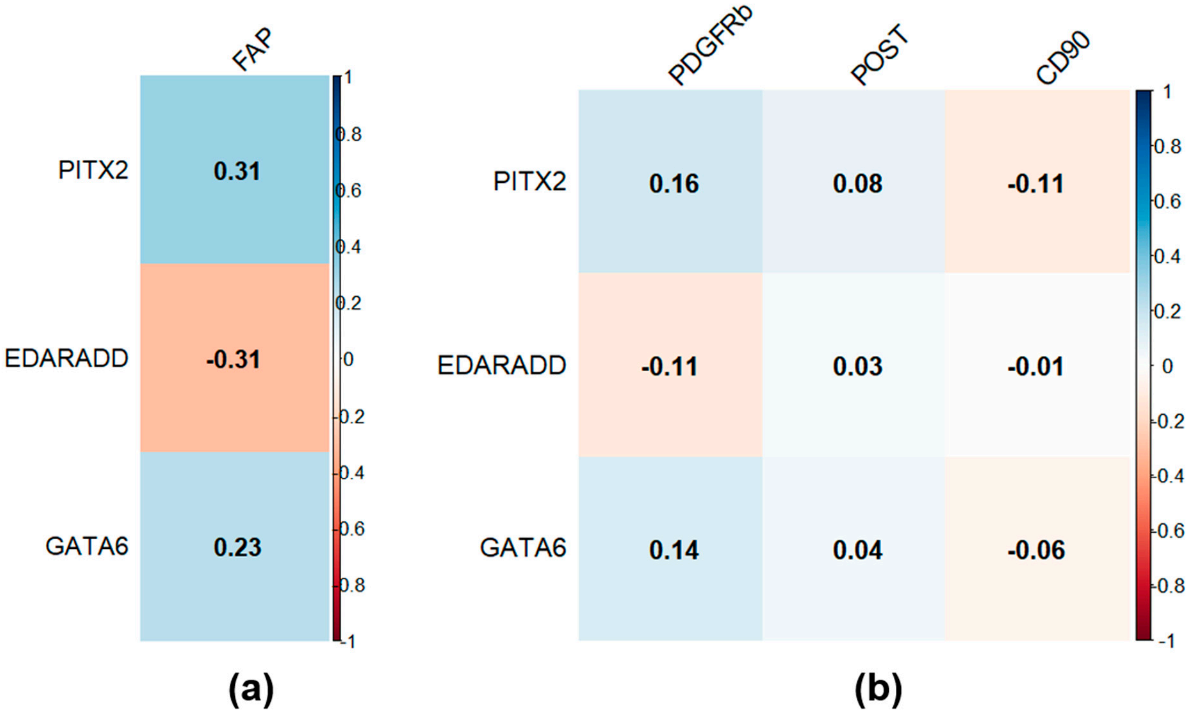

Figure S2. Correlation matrix for the studied CAF markers in PC samples. Significant results at $p<0.05$ are absent. a – correlation matrix for FAP (separate due to low sample size), b – correlation matrix for PDGFRb, POST, and CD90.
