# Supplementary material for: Prognostic Potential of Cancer-Associated Fibroblast Surface Markers and Their Specific DNA Methylation in Prostate Cancer
Source: Diagnostics (Basel). 2025 Sep 24;15(19):2434. doi: 10.3390/diagnostics15192434 (PMC12524081; doi:10.3390/diagnostics15192434)
Supplement: Supplementary file 1 [file diagnostics-15-02434-s001.zip › Table S2.pdf]

Table S2. Sequences of oligonucleotide primers and probes used in methylation-specific qPCR and ddPCR assays.

Gene	Oligonucleotide	Sequence
EDARADD	Meth. forward primer	5'-AGA TAA TTA GCG AGT ATT TTT TCG T-3'
	Meth. reverse primer	5'-ATA CCT CTC CCC ATC TAT TTA ATC G-3'
	Unmeth. forward primer	5'-AGA TAA TTA GTG AGT ATT TTT TTG T-3'
	Unmeth. reverse primer	5'-ATA CCT CTC CCC ATC TAT TTA ATC AT-3'
GATA6	Meth. forward primer	5'-GTT TTG TTT TTA GGA TTT TTT CGT-3'
	Meth. reverse primer	5'-CAA ATA TAC ATT TAA TCT CAT CCG TA-3'
	Unmeth. forward primer	5'-GTT TTG TTT TTA GGA TTT TTT TGT-3'
	Unmeth. reverse primer	5'-CAA ATA TAC ATT TAA TCT CAT CCA TA-3'
PITX2[1]	Forward primer	5'-GTA GGG GAG GGA AGT AGA TGT T-3'
	Reverse primer	5'-TTC TAA TCC TCC TTT CCA CAA TAA-3'
	Meth. probe	5'-FAM-AGT CGG AGT CGG GAG AGC GA-BHQ1-3'
ACTB	Forward primer	5'-TGG TGA TGG AGG AGG TTT AGT AAG T-3'
	Reverse primer	5'-AAC CAA TAA AAC CTA CTC CTC CCT TAA-3'
	Meth. probe	5'-HEX-ACC ACC ACC CAA CAC ACA ATA ACA AAC ACA-BHQ1-3'
ACTB[2]	Forward primer	5'-GCG CCG TTC CGA AAG TT-3'
	Reverse primer	5'-CGG CGG ATC GGC AAA-3'
	Meth. probe	5'-HEX-ACC GCC GAG ACC GCG TC-BHQ1-3'

HEX, Hexachlorofluorescein; FAM, 6-Carboxyfluorescein; BHQ1, Black Hole Quencher 1. [1] Sequences of oligonucleotides for the PITX2 gene were obtained from a study conducted by Weiss et al. (DOI:10.1016/j.juro.2008.11.120). [2] Was used only to confirm the completeness of

the bisulfite conversion, qPCR conditions were the same as for ACTB (see Materials and Methods section of the manuscript).
