# Supplementary material for: Prognostic Potential of Cancer-Associated Fibroblast Surface Markers and Their Specific DNA Methylation in Prostate Cancer
Source: Diagnostics (Basel). 2025 Sep 24;15(19):2434. doi: 10.3390/diagnostics15192434 (PMC12524081; doi:10.3390/diagnostics15192434)
Supplement: Supplementary file 1 [file diagnostics-15-02434-s001.zip › Table S3.pdf]

Table S3. The clinical and morphological features compared between groups with different CAF markers expression (data were obtained based on the immunohistochemical analysis results).

	**FAP**			**PDGFRb**			**POST**			**CD90**		
	No n=18	Yes n=16	p	Low n=57	High n=31	p	Low n=31	High n=57	p	Low n=66	High n=20	p
Age, years, median (Q1-Q3)	64.0 (61.0-68.8)	63.5 (61.3-66.0)	0.931	64.0 (59.0-68.0)	64.0 (61.0-69.0)	0.632	63.5 (60.0-69.0)	64.0 (61.0-68.0)	0.570	64.0 (60.0-69.0)	64.0 (60.8-68.3)	0.752
PSA, ng/ml, median (Q1-Q3)	6.7 (4.8-15.6)	10.9 (6.3-16.6)	0.211	7.0 (5.2-12.0)	7.7 (5.1-16.5)	0.407	6.7 (4.7-14.8)	7.7 (6.2-13.3)	0.339	6.8 (5.1-10.8)	12.0 (6.6-19.0)	0.009*
MRI lesion, % (n)	77.8% (14)	93.8% (15)	0.340	79.0% (45)	93.6% (29)	0.125	83.9% (26)	84.2% (48)	1.000	81.8% (54)	90.0% (18)	0.505
Gleason score, median (Q1-Q3)	7.0 (6.0-7.0)	7.5 (7.0-8.0)	0.016*	7.0 (6.0-7.0)	7.0 (7.0-7.0)	0.003*	7.0 (6.0-7.0)	7.0 (7.0-7.0)	0.103	7.0 (7.0-7.0)	7.0 (7.0-8.0)	0.036*
Gleason			0.014*			0.016*			0.504			0.029*
• 3+4=7 and less, % (n)	66.7% (12)	18.8% (3)		76.8% (43)	48.3% (14)		73.3% (22)	63.6% (35)		74.6% (47)	45.0% (9)	
• 4+3=7 and more, % (n)	33.3% (6)	81.3% (13)		23.2% (13)	51.7% (15)		26.7% (8)	36.4% (20)		25.4% (16)	55.0% (11)	
pT stage			0.162			0.207			0.474			0.222
• pT2, % (n)	61.1% (11)	31.3% (5)		73.7% (42)	58.1% (18)		74.2% (23)	64.9% (37)		72.7% (48)	55.0% (11)	
• pT3, % (n)	38.9% (7)	68.8% (11)		26.3% (15)	41.9% (13)		25.8% (8)	35.1% (20)		27.3% (18)	45.0% (9)	
pN stage			0.078			0.232			0.219			0.669
• 0, % (n)	94.4% (17)	68.8% (11)		94.6% (53)	86.7% (26)		86.2% (25)	94.7% (54)		92.2% (59)	90.0% (18)	
• 1, % (n)	5.6% (1)	31.3% (5)		5.4% (3)	13.3% (4)		13.8% (4)	5.3% (3)		7.8% (5)	10.0% (2)	
Pn, % (n)	83.3% (15)	81.3% (13)	1.000	80.7% (46)	90.3% (28)	0.362	74.2% (23)	89.5% (51)	0.074	83.3% (55)	90.0% (18)	0.724
LI, % (n)	5.6% (1)	43.8% (7)	0.014*	22.8% (13)	29.0% (9)	0.699	25.8% (8)	24.6% (14)	0.897	21.2% (14)	35.0% (7)	0.241

The significance levels below 0.05 are marked with "*". PSA, prostate specific antigen; MRI, magnetic resonance imaging; LI, perilymphatic invasion.
