# Supplementary material for: Prognostic Potential of Cancer-Associated Fibroblast Surface Markers and Their Specific DNA Methylation in Prostate Cancer
Source: Diagnostics (Basel). 2025 Sep 24;15(19):2434. doi: 10.3390/diagnostics15192434 (PMC12524081; doi:10.3390/diagnostics15192434)
Supplement: Supplementary file 1 [file diagnostics-15-02434-s001.zip › Table S4.pdf]

Table S4. The expression of CAF markers in different clinical and morphological groups (data were obtained based on the immunohistochemical analysis results).

	**FAP expression**	**p**	**PDGFRb expression**	**p**	**POST expression**	**p**	**CD90 expression**	**p**
PSA		0.265		0.063		0.963		0.021*
• Below 10 ng/ml, (n=57)	0.0 (0.0-1.0)		2.0 (1.0-3.0)		2.0 (1.0-3.0)		0.0 (0.0-1.0)	
• 10 ng/ml and more, (n=31)	1.0 (0.0-1.8)		2.0 (1.0-3.0)		2.0 (1.0-3.0)		1.0 (0.0-2.0)	
MRI lesion		0.165		0.139		0.899		0.973
• No	0.0 (0.0-0.0)		1.0 (1.0-2.0)		2.0 (1.0-3.0)		0.0 (0.0-1.0)	
• Yes	1.0 (0.0-1.0)		2.0 (1.0-3.0)		2.0 (1.0-3.0)		0.0 (0.0-1.3)	
Gleason		0.006*		0.001*		0.937		0.031*
• 3+4=7 and less	0.0 (0.0-0.0)		1.0 (1.0-2.0)		2.0 (1.0-3.0)		0.0 (0.0-1.0)	
• 4+3=7 and more	1.0 (0.0-2.0)		3.0 (2.0-3.0)		2.0 (1.0-3.0)		1.0 (0.0-3.0)	
pT stage		0.155		0.024*		0.977		0.991
• pT2, (n=60)	0.0 (0.0-1.0)		2.0 (1.0-3.0)		2.0 (1.0-3.0)		0.0 (0.0-1.0)	
• pT3, (n=28)	1.0 (0.0-1.0)		2.0 (2.0-3.0)		2.0 (1.0-3.0)		0.0 (0.0-2.0)	
pN stage		0.026*		0.204		0.186		0.596
• 0, % (n), (n=79)	0.0 (0.0-1.0)		2.0 (1.0-3.0)		2.0 (1.0-3.0)		0.0 (0.0-1.0)	
• 1, % (n), (n=7)	1.5 (1.0-2.0)		3.0 (2.0-3.0)		1.0 (0.5-2.5)		0.0 (0.0-1.0)	
Pn		1.000		0.144		0.097		0.135
• No, (n=14)	0.5 (0.0-1.0)		1.0 (1.0-2.0)		1.0 (1.0-2.8)		0.0 (0.0-0.0)	
• Yes, (n=74)	0.0 (0.0-1.0)		2.0 (1.0-3.0)		2.0 (1.0-3.0)		0.0 (0.0-1.0)	
LI		0.005*		0.214		0.535		0.066
• No, (n=66)	0.0 (0.0-1.0)		2.0 (1.0-3.0)		2.0 (1.0-3.0)		0.0 (0.0-1.0)	
• Yes, (n=22)	1.5 (1.0-2.0)		2.0 (1.3-3.0)		2.0 (1.0-3.0)		1.0 (0.0-2.0)	

The significance levels below 0.05 are marked with "*". PSA, prostate specific antigen; MRI, magnetic resonance imaging; LI, perilymphatic invasion.
