# Supplementary material for: Prognostic Potential of Cancer-Associated Fibroblast Surface Markers and Their Specific DNA Methylation in Prostate Cancer
Source: Diagnostics (Basel). 2025 Sep 24;15(19):2434. doi: 10.3390/diagnostics15192434 (PMC12524081; doi:10.3390/diagnostics15192434)
Supplement: Supplementary file 1 [file diagnostics-15-02434-s001.zip › Table S5.pdf]

Table S5. The clinical and morphological features compared between groups with different DNA methylation profiles (qPCR).

	**PITX2 methylation level**			**EDARADD methylation level**			**GATA6 methylation level**		
	Low n=64	High n=13	p	Low n=38	High n=37	p	Low n=40	High n=40	p
Age, years, median (Q1-Q3)	64.5 (61.0-69.0)	63.0 (60.0-65.0)	0.169	63.0 (60.3-68.8)	65.0 (62.0-69.0)	0.339	65.0 (60.0-70.0)	64.0 (60.8-68.3)	0.626
BMI, kg/m^2, median (Q1-Q3)	27.2 (25.7-29.8)	26.6 (22.4-28.8)	0.153	27.2 (25.3-29.9)	27.2 (25.5-29.2)	0.814	28.7 (25.9-30.4)	26.8 (24.8-28.9)	0.052
PSA, ng/ml, median (Q1-Q3)	7.8 (5.2-13.6)	6.7 (5.2-13.6)	0.744	7.0 (5.4-13.0)	8.7 (5.2-13.7)	0.741	7.0 (5.2-12.0)	8.4 (5.6-14.4)	0.436
MRI lesion, % (n)	82.8% (53)	100% (13)	0.194	84.2% (32)	83.8% (31)	0.791	77.5% (31)	92.5% (37)	0.117
Prostate volume, cm^3, median (Q1-Q3)	28.9 (30.0-51.5)	30.0 (28.3-36.3)	0.114	35.0 (29.4-50.0)	37.7 (28.5-47.8)	0.925	36.2 (30.0-68.5)	36.5 (29.0-44.3)	0.417
Gleason score, median (Q1-Q3)	7.0 (7.0-7.0)	7.0 (7.0-7.0)	0.748	7.0 (7.0-7.0)	7.0 (7.0-7.0)	0.947	7.0 (7.0-7.0)	7.0 (7.0-7.0)	0.540
Gleason			0.219			0.768			0.490
• 3+4=7 and less, % (n)	65.6% (40)	46.2% (6)		62.9% (22)	59.5% (22)		67.6% (25)	60.0% (24)	
• 4+3=7 and more, % (n)	34.4% (21)	53.8% (7)		37.1% (13)	40.5% (15)		32.4% (12)	40.0% (16)	
pT stage			0.052			0.744			0.813
• pT2, % (n)	70.3% (45)	38.5% (5)		65.8% (25)	62.2% (23)		67.5% (27)	65.0% (26)	
• pT3, % (n)	29.7% (19)	61.5% (8)		34.2% (13)	37.8% (14)		32.5% (13)	25.0% (14)	
pN stage, % (n)			1.000			1.000			1.000
• 0	92.1% (58)	91.7% (11)		91.7% (33)	91.9% (34)		92.1% (35)	92.5% (37)	
• 1	7.9% (5)	8.3% (1)		8.3% (3)	8.11% (3)		7.9% (3)	7.5% (3)	
Pn, % (n)	81.3% (52)	92.3% (12)	0.449	81.6% (31)	83.8% (31)	0.958	82.5% (33)	85.0% (34)	1.000
LI, % (n)	21.9% (14)	46.2% (6)	0.088	21.1% (8)	32.4% (12)	0.394	27.5% (11)	22.5% (9)	0.606
Recurrence, % (n)	23.1% (12)	33.3% (3)	0.676	25.8% (8)	20.7% (6)	0.871	17.1% (6)	31.0% (9)	0.313

The significance levels below 0.05 are marked with "*". PSA, prostate specific antigen; MRI, magnetic resonance imaging; LI, perilymphatic invasion.
