# Supplementary material for: Prognostic Potential of Cancer-Associated Fibroblast Surface Markers and Their Specific DNA Methylation in Prostate Cancer
Source: Diagnostics (Basel). 2025 Sep 24;15(19):2434. doi: 10.3390/diagnostics15192434 (PMC12524081; doi:10.3390/diagnostics15192434)
Supplement: Supplementary file 1 [file diagnostics-15-02434-s001.zip › Table S6.pdf]

Table S6. The qPCR DNA methylation levels for PITX2, EDARADD and GATA6 in different clinical and morphological groups

	**PITX2 methylation level**	**p**	**EDARADD methylation level**	**p**	**GATA6 methylation level**	**p**
PSA		0.741		0.500		0.772
• Below 10 ng/ml, (n=57)	0.0 (0.0-0.0)		84.0 (73.6-92.7)		81.7 (68.5-89.4)	
• 10 ng/ml and more, (n=31)	0.0 (0.0-0.0)		88.8 (80.0-94.0)		83.7 (71.8-89.6)	
MRI lesion		0.113		0.423		0.159
• No, (n=14)	0.0 (0.0-0.0)		84.5 (73.3-91.9)		72.6 (65.3-83.3)	
• Yes, (n=74)	0.0 (0.0-0.0)		85.5 (76.2-94.0)		83.1 (73.0-89.7)	
Gleason		0.109		0.169		0.904
• 3+4=7 and less, (n=57)	0.0 (0.0-0.0)		85.7 (74.7-92.4)		81.9 (71.8-90.3)	
• 4+3=7 and more, (n=28)	0.0 (0.0-7.1)		88.5 (83.3-94.4)		83.2 (72.6-88.5)	
pT stage		0.026*		0.705		0.583
• pT2, (n=60)	0.0 (0.0-0.0)		84.8 (75.4-93.0)		81.9 (68.3-87.9)	
• pT3, (n=28)	0.0 (0.0-19.4)		87.3 (73.3-93.5)		82.8 (72.9-90.9)	
pN stage		0.915		0.756		0.715
• 0, (n=79)	0.0 (0.0-0.0)		87.3 (78.1-93.5)		82.8 (71.1-89.7)	
• 1, (n=7)	0.0 (0.0-0.0)		81.2 (66.4-94.7)		78.4 (59.9-90.3)	
Pn		0.302		0.721		0.611
• No, (n=14)	0.0 (0.0-0.0)		79.8 (73.0-94.2)		78.1 (67.8-86.8)	
• Yes, (n=74)	0.0 (0.0-0.0)		86.4 (77.7-93.0)		82.7 (71.6-90.1)	
LI		0.071		0.619		0.289
• No, (n=66)	0.0 (0.0-0.0)		84.1 (76.2-94.0)		82.8 (73.0-89.9)	
• Yes, (n=22)	0.0 (0.0-11.9)		88.1 (70.4-92.0)		76.9 (65.3-88.5)	
Recurrence		0.489		0.993		0.561
• No, (n=53)	0.0 (0.0-0.0)		86.4 (74.0-92.5)		79.3 (68.7-89.8)	
• Yes, (n=15)	0.0 (0.0-0.0)		84.1 (74.1-93.5)		83.7 (76.1-86.3)	

The significance levels below 0.05 are marked with “*”. PSA, prostate specific antigen; MRI, magnetic resonance imaging; LI, perilymphatic invasion.
