# Supplementary material for: Prognostic Potential of Cancer-Associated Fibroblast Surface Markers and Their Specific DNA Methylation in Prostate Cancer
Source: Diagnostics (Basel). 2025 Sep 24;15(19):2434. doi: 10.3390/diagnostics15192434 (PMC12524081; doi:10.3390/diagnostics15192434)
Supplement: Supplementary file 1 [file diagnostics-15-02434-s001.zip › Table S7.pdf]

Table S7. The clinical and morphological features compared between groups with different DNA methylation profiles

	**PITX2 methylation level**			**EDARADD methylation level**			**GATA6 methylation level**		
	**Low** n=39	**High** n=40	**p**	**Low** n=39	**High** n=40	**p**	**Low** n=40	**High** n=39	**p**
Age, years, median (Q1-Q3)	64.0 (59.0-68.0)	65.0 (62.8-69.3)	0.109	63.0 (60.5-67.5)	66.0 (60.8-72.0)	0.195	66.0 (60.8-70.5)	63.0 (60.5-67.5)	0.157
BMI, kg/m^2, median (Q1-Q3)	27.5 (25.6-29.8)	27.0 (25.5-29.0)	0.405	27.2 (25.7-29.1)	27.2 (25.4-29.8)	0.996	27.1 (25.4-29.4)	27.3 (25.7-29.7)	0.691
PSA, ng/ml, median (Q1-Q3)	7.0 (5.3-11.9)	7.7 (5.2-13.9)	0.556	7.7 (4.9-15.2)	7.1 (5.3-10.4)	0.765	7.6 (5.6-11.3)	7.2 (4.8-15.3)	0.910
MRI lesion, % (n)	74.4% (29)	95.0% (38)	0.025*	87.2% (34)	82.5% (33)	0.790	80.0% (32)	89.7% (35)	0.372
Prostate volume, cm^3, median (Q1-Q3)	40.0 (31.0-55.8)	34.0 (28.0-44.8)	0.076	35.0 (28.9-46.0)	39.3 (30.0-55.0)	0.477	36.6 (28.8-53.5)	36.0 (30.0-48.0)	0.932
Gleason score, median (Q1-Q3)	7.0 (7.0-7.0)	7.0 (7.0-7.0)	0.130	7.0 (7.0-7.0)	7.0 (7.0-7.0)	0.711	7.0 (7.0-7.0)	7.0 (7.0-7.0)	0.995
Gleason			0.028*			0.154			0.211
• 3+4=7 and less, % (n)	75.7% (28)	51.3% (20)		55.3% (21)	71.1% (27)		70.3% (26)	56.4% (22)	
• 4+3=7 and more, % (n)	24.3% (32)	48.7% (19)		44.7% (17)	29.0% (11)		29.7% (11)	43.6% (17)	
pT stage			0.040*			0.750			0.428
• pT2, % (n)	76.9% (30)	55.0% (22)		64.1% (25)	67.5% (27)		70.0% (28)	61.5% (24)	
• pT3, % (n)	23.1% (9)	45.0% (18)		35.9% (14)	32.5% (13)		30.0% (12)	38.5% (15)	
pN stage			0.200			0.675			0.675
• 0, % (n)	97.4% (37)	87.2% (34)		89.7% (35)	94.7% (36)		94.7% (36)	89.7% (35)	
• 1, % (n)	2.6% (1)	12.8% (5)		10.3% (4)	5.3% (2)		5.3% (2)	10.3% (4)	
Pn, % (n)	79.5% (31)	87.5% (35)	0.511	79.5% (31)	87.5% (35)	0.511	85.0% (34)	82.1% (32)	0.960
LI, % (n)	20.5% (8)	30.0% (12)	0.477	33.3% (13)	17.5% (7)	0.174	22.5% (9)	28.2% (11)	0.746

The significance levels below 0.05 are marked with "*". PSA, prostate specific antigen; MRI, magnetic resonance imaging; LI, perilymphatic invasion.
