# Supplementary material for: Prognostic Potential of Cancer-Associated Fibroblast Surface Markers and Their Specific DNA Methylation in Prostate Cancer
Source: Diagnostics (Basel). 2025 Sep 24;15(19):2434. doi: 10.3390/diagnostics15192434 (PMC12524081; doi:10.3390/diagnostics15192434)
Supplement: Supplementary file 1 [file diagnostics-15-02434-s001.zip › Table S8.pdf]

Table S8. The ddPCR DNA methylation levels for PITX2, EDARADD and GATA6 in different clinical and morphological groups

	**PITX2 methylation level**	**p**	**EDARADD methylation level**	**p**	**GATA6 methylation level**	**p**
PSA		0.196		0.279		0.205
• Below 10 ng/ml, (n=57)	3.8 (1.5-7.8)		87.9 (65.3-100.0)		73.0 (44.6-81.1)	
• 10 ng/ml and more, (n=31)	6.2 (2.8-11.1)		66.4 (55.6-100.0)		77.8 (52.2-82.9)	
MRI lesion		0.017*		0.557		0.108
• No, (n=14)	2.6 (1.1-3.9)		91.5 (64.4-100.0)		49.1 (24.4-79.7)	
• Yes, (n=74)	6.1 (1.8-8.7)		78.1 (56.9-100.0)		74.8 (49.7-82.6)	
Gleason		0.054		0.203		0.065
• 3+4=7 and less, (n=57)	3.8 (1.6-7.1)		89.8 (62.8-100.0)		72.8 (45.4-79.7)	
• 4+3=7 and more, (n=28)	6.9 (2.7-12.3)		70.2 (57.2-98.2)		81.3 (52.8-84.7)	
pT stage		0.025*		0.783		0.171
• pT2, (n=60)	3.8 (1.5-7.1)		81.0 (55.9-100.0)		73.0 (44.0-81.4)	
• pT3, (n=28)	7.2 (3.4-12.9)		77.2 (63.5-98.8)		77.5 (67.9-83.9)	
pN stage		0.408		0.197		0.356
• 0, % (n), (n=79)	4.2 (1.7-8.3)		78.4 (60.0-100.0)		74.3 (46.1-82.1)	
• 1, % (n), (n=7)	6.7 (5.5-8.2)		62.6 (52.8-86.5)		79.9 (72.2-83.2)	
Pn		0.202		0.270		0.275
• No, (n=14)	2.8 (1.5-6.1)		65.6 (51.0-100.0)		75.0 (69.6-83.7)	
• Yes, (n=74)	5.0 (1.9-8.7)		85.1 (64.7-100.0)		73.7 (43.1-81.7)	
LI		0.201		0.161		0.234
• No, (n=66)	3.8 (1.3-7.8)		89.2 (65.3-100.0)		73.4 (42.4-81.6)	
• Yes, (n=22)	6.2 (3.0-8.6)		65.5 (55.3-95.9)		78.1 (68.1-83.8)	

The significance levels below 0.05 are marked with "*". PSA, prostate specific antigen; MRI, magnetic resonance imaging; LI, perilymphatic invasion.
