# Supplementary material for: Prognostic Potential of Cancer-Associated Fibroblast Surface Markers and Their Specific DNA Methylation in Prostate Cancer
Source: Diagnostics (Basel). 2025 Sep 24;15(19):2434. doi: 10.3390/diagnostics15192434 (PMC12524081; doi:10.3390/diagnostics15192434)
Supplement: Supplementary file 1 [file diagnostics-15-02434-s001.zip › Table S9.pdf]

Table S9. DNA methylation levels depending on the expression of different CAF markers

	PITX2, %, median (Q1-Q3)	p	EDARADD, %, median (Q1-Q3)	p	GATA6, %, median (Q1-Q3)	p
FAP		0.119		0.348		0.318
• No (n=16)	2.6 (1.3-4.8)		81.0 (65.8-100.0)		73.8 (34.1-79.9)	
• Yes (n=16)	6.9 (2.1-9.3)		68.4 (57.3-98.2)		79.9 (66.7-84.3)	
PDGFRb		0.027*		0.208		0.144
• Low (n=50)	3.8 (1.5-6.7)		87.9 (65.2-100.0)		71.3 (45.2-80.1)	
• High (n=29)	7.5 (3.5-11.6)		75.3 (51.5-95.3)		78.4 (65.3-84.2)	
POST		0.319		0.586		0.360
• Low (n=27)	3.8 (1.8-7.1)		78.3 (55.8-100.0)		74.3 (45.0-79.7)	
• High (n=52)	5.0 (1.7-8.6)		83.8 (64.0-100.0)		74.1 (46.6-84.1)	
CD90		0.563		0.537		0.566
• Low	5.0 (1.9-8.0)		85.1 (64.9-100.0)		73.0 (48.5-80.2	
• High	3.8 (1.2-9.5)		77.2 (50.8-100.0)		78.4 (30-.7-84.2)	

The significance levels below 0,05 are marked with “*”.
